# Out-of-pocket pharmaceutical expenditure and potential misuse of public resources - analysis in the Italian context

**DOI:** 10.1186/s12962-025-00619-7

**Published:** 2025-04-17

**Authors:** Leonarda Maurmo, Federico Ruta, Grazia Dicuonzo, Vincenzo Signoretta, Cosimo Gennari, Vincenzo Dicuonzo, Donato Suma, Mariarosaria D’Ambrosio, Cataldo Procacci

**Affiliations:** 1 University of Bari Aldo Moro, Bari, Italy; 2 Health Agency BAT, Andria, Italy; 3 Pharmaceutical Assistance Management AUSL Imola, Imola, Italy; 4 Hsptl, Suppl Svc Expert, ITA Strategy & Operations, IQVIA, Milan, Italy; 5 Catholic University Our Lady of Good Counsel, Tirana, Albania; 6 Pharmaceutical Department, Health Agency BAT, Andria, Italy; 7 Pharmacist, Italy

**Keywords:** Private health care expenditure, Prescribing appropriateness, Territorial pharmaceuticals

## Abstract

The increase in longevity determines a greater need to receive medical care and pharmacological treatments. The introduction of patient cost-sharing in pharmaceutical expenditures aims to finance the National/Regional Health System while simultaneously reducing excessive consumption of health services. However, in the context of national health insurance, decreasing drug expenditures can lead to increased hospitalization costs. This phenomenon highlights the complexity of healthcare financing, where measures intended to control spending in one area may inadvertently escalate costs in another, necessitating a careful evaluation of health policies and their broader implications on patient care and system sustainability. The main focus of the analysis is to examine drug expenditures and the private purchase of drugs. Specifically, the analysis investigates the spending on drug therapies across different regional macro-areas (the Italian regional macro-areas are geographical subdivisions used to organize activities and services, particularly in the context of healthcare and scientific research.). Moreover, it analized the variability in the use of AIFA (Italian Medicines Agency) notes, a regulatory tool used in Italy to define the reimbursement criteria and therapeutic indications for which a drug can be prescribed at the expense of the National Health Service (SSN), and the extent to which individuals resort to private purchasing for drugs that are in total Health Service reimbursement. Additionally, the analysis delves into the top 30 active substances that significantly impact pharmaceutical spending, as reported in the Osmed 2022 report. This research found heterogeneous use of AIFA notes for many drugs across Italy. Inappropriate use of restrictive notes at prescription indicates high patient out-of-pocket spending, constituting financial damage. Comparing regional ratios to national benchmarks reveals deviations in prescribing behavior and AIFA note use by GPs. Regions with highest inappropriate AIFA note use also have highest out-of-pocket spending and lowest incomes, suggesting cultural factors drive branded over generic drug choices when public reimbursement is available.There can be many causes, including a cultural nature, which push patients to purchase the originator drug by paying the excess amount.

**Supplementary Information:**

The online version contains supplementary material available at 10.1186/s12962-025-00619-7.

## Introduction

Italian health spending shows an almost negligible real growth rate in the decade 2010–2019 (+ 0.4%), compared with EU countries increased by an annual average (median) of 2.6% a year.

This grow rate is also lower compared to the rate recorded in the decade 2000–2010 (2.0%). Its composition is an important aspect of health spending in relation to the paying subjects: being a critical sector in every country both socially and economically, healthcare always has a strong presence of the public sector, although with variable objectives and scope [1, 2].

The Italian healthcare expenditure (2,686 € per inhabitant) is lower than the European average value (3,269 € per inhabitant) [3], representing the 9,6% of GPD instead of the European average percentage of 10,9% of GPD.

The Budget Law for 2022 (Law No. 234/2021) has set the total funding for public healthcare and accredited healthcare in Italy at 124.061 million euros for 2022, 126.061 million for 2023, and 128.061 million for the year 2024.

Although Italy guarantees public healthcare coverage through the National Health Service at 70%, the out-of-pocket expenditure in our country is approximately 23%, which is above the European average of around 16% [4–5].

In 2019, public funding for health in Italy was 73.9%, exceeding the EU average (71.7%). The remaining funding was primarily from private sources, with out-of-pocket (OOP) spending accounting for 23.3% and voluntary health insurance (VHI) contributing only 2.1%. Total private expenditure reached approximately EUR 40 billion, with EUR 35.8 billion in OOP costs and EUR 4.3 billion for VHI, occupational medicine, and non-profit services. In 2020, OOP expenditure decreased to EUR 33.9 billion, while other components remained stable [6].

It should be considered that:

- 40% of out-of-pocket expenses in Italy are allocated to outpatient medical care;
- Nearly half of this expenditure is dedicated to dental care;
- Outpatient pharmaceutical products, on the other hand, represent approximately 30% of the total out-of-pocket expenses.

The proportion between the contributions of the three payers (public sector, insurance, and citizens-patients) can be seen as a measure of equity in the healthcare system [7]. In fact, a high share of public spending corresponds to a strong wealth redistribution action, contributing to making the system more equitable in access to services.

The out-of-pocket expenses, which make up 23.3% of healthcare spending in Italy, are costs that patients directly incur at the point of service use. These costs are regressive in relation to income, meaning they disproportionately affect those with lower incomes. This category includes expenses borne by individuals who have either been unable or chosen not to secure private insurance coverage.

Private healthcare spending is intimately tied to an individual’s financial capacity. A system that relies heavily on private insurance spending can create access barriers for those who cannot afford coverage, are not eligible for public programs, or whose employers do not provide health insurance. Cultural factors and misconceptions about the extent of public coverage can also create access barriers and hinder the adoption of supplementary insurance schemes.

The provided search results offer insights into Italy’s healthcare expenditure, particularly the distribution of public and private spending. The data highlights the significant out-of-pocket spending in Italy, which accounts for 23% of total healthcare expenditure, indicating a substantial financial burden on individuals at the time of consumption.

A recent study on out-of-pocket spending for the treatment of chronic intestinal inflammations suggests that in Italy, increases in out-of-pocket expenses could be seen as a response from patients aiming to compensate for deficiencies and inefficiencies in public healthcare provision, concluding that the increase in this expenditure category should be considered as an indicator of poor quality of care and difficulties in accessing treatment [8].

The role of cost-sharing could be particularly relevant in countries where public spending is increasingly subject to budget constraints and where, as a generalized trend, public coverage has been reduced in recent years. In such circumstances, taking into account the different determinants of public and private consumption choices, cost-sharing can conceptually evolve from a mere substitute for public spending to a contribution for qualitative upgrades of services. In this sense, rather than representing an inequitable tool leading to the renunciation of care, cost-sharing could be one of the primary drivers for the dissemination of new services and technologies, thereby freeing up public resources for essential and priority services.

Traditional approaches to analyzing healthcare spending are based on the assumption that the total amount of healthcare expenditure (public, out-of-pocket, and intermediated) results in an improvement in individual and collective health. However, if value is the ratio between health outcomes relevant to the patient and costs, waste and inefficiencies that consume resources without generating value reduce the value for money, which is the return in terms of health for the resources invested in healthcare [9, 10]. This includes improper resource allocation (fraud and abuse), purchasing costs exceeding the value of the product, administrative inefficiencies (excessive bureaucracy, inadequate digitization), inadequate coordination between different care settings, and low productivity. It also encompasses the delivery of ineffective or inappropriate healthcare interventions with low or negative value, as well as the consequences of not providing effective and appropriate healthcare interventions with high value [11].

Furthermore, the 2022 OASI Report [12], based on a reanalysis of data from the OECD Health Statistics 2022, has highlighted in recent years that Italy, despite having a universal healthcare system, ranks among countries with the lowest per capita healthcare expenditure compared to other advanced countries. In 2019, public healthcare spending in Germany, France, and the United Kingdom was at least 50% higher than in Italy. Moreover, when compared to per capita private spending, Italy ranks below these three countries and even below Spain and Portugal. This positioning is also confirmed when considering the data relative to GDP, indicating that Italy allocates a lower percentage of its GDP to healthcare spending compared to other countries.

In Italy, pharmaceutical expenditure constitutes a significant portion of total healthcare spending. In 2022, total healthcare expenditure reached approximately €170 billion, with pharmaceutical expenditure amounting to around €34.1 billion, reflecting a 6% increase compared to the previous year. This data highlights the ongoing growth in pharmaceutical costs within the broader context of healthcare financing in Italy.

The share of pharmaceutical spending within the overall healthcare expenditure in Italy was approximately 76.4% covered by the National Health Service (SSN) in 2019. This figure highlights the significant role that public funding plays in pharmaceutical expenditures compared to private sources [13].

### Overview of the Italian National health system: promoting prescribing appropriateness and medication reimbursement classes

The Italian National Health System of the Beveridge model promotes prescribing appropriateness by guiding the use of medications towards more effective and safer therapeutic indications [14]. This is achieved through tools such as AIFA Notes, which define the indications for which a drug is reimbursable by the National Health Service (SSN), and Therapeutic Plans, which provide evidence-based guidelines. Furthermore, monitoring pharmaceutical consumption is essential to ensure responsible use of resources.

The Beveridge system ensures universal access to essential medications while promoting appropriate and responsible use of pharmaceutical resources through public governance involving institutions like AIFA, thus contributing to the sustainability and effectiveness of the healthcare system.

In Italy, the reimbursement classes for medications are categorized primarily into three main groups, which determine how costs are covered by the National Health Service (SSN):

Class A: This class includes essential medications that are fully reimbursed by the SSN. These drugs are considered necessary for public health and are prescribed for a wide range of conditions. Patients typically do not pay out-of-pocket for these medications, although some regions may impose a nominal ticket fee.

Class H: This category encompasses medications that are used exclusively in hospitals. These drugs are also fully reimbursed by the SSN and are administered in healthcare facilities.

Class C: Medications in this class are not reimbursed by the SSN and are typically paid for entirely by the patient. However, some drugs in this category may require a prescription, and healthcare providers are encouraged to inform patients about equivalent medications that may be less expensive.

AIFA Notes are regulatory tools that define the therapeutic indications for which a drug is reimbursable by the National Health Service (SSN) in Italy. These notes can be introduced in specific circumstances, such as when a drug is authorized for different clinical indications or when it is intended to prevent significant risks for particular population groups. Some drugs, although initially covered by a Note, may be excluded from reimbursement and classified in Class C, at the expense of the citizen. In this case, the patient must pay the full cost of the drug, unless there are cheaper generic alternatives available. AIFA Notes are regularly updated to reflect new scientific evidence and ensure appropriate use of pharmaceutical resources.

Another type of out-of-pocket drug expenditure is the co-payment system for brand-name drugs.

The co-payment system for brand-name drugs in Italy requires patients to contribute financially to the purchase of medications, in addition to the reimbursement provided by the National Health Service (SSN). This mechanism aims to manage healthcare costs and promote responsible use of pharmaceuticals, but it can lead to significant expenses for patients who prefer brand-name options over equivalent generics. Co-payment varies based on the reimbursement class of the drug, the patient’s income, and regional regulations, with differences in implementation at the local level. While co-payment may influence patients’ choices towards generics, some still prefer brand-name medications due to perceived quality. The Italian government is working to promote the use of equivalent medications through informational campaigns to reduce overall healthcare spending and improve access to essential treatments.

The aim of this study is to assess inter-regional differences in out-of-pocket spending and the different utilization of AIFA Notes.

## Materials and methods

An analysis was conducted by consulting the administrative databases. First, the private component of pharmaceutical expenditure was verified using the OsMed (Osservatorio Nazionale sull’Impiego dei Medicinali) Report 2022, this report is an annual publication by the Italian Medicines Agency (AIFA) that provides comprehensive data and analysis on the use of medicines in Italy.

The OsMed report, officially titled “Medicines Use in Italy,” is a comprehensive analysis produced by the National Observatory on the Use of Medicines (OsMed) under the Italian Medicines Agency (AIFA). This report, now in its twenty-first edition, provides critical insights into pharmaceutical care within Italy, detailing drug utilization, expenditure, and the impact of various healthcare policies. It encompasses data on both inpatient and outpatient settings, highlighting trends in prescription practices and comparing Italian data with those from other European countries. The OsMed report serves as a vital resource for understanding the dynamics of medicine usage in Italy and informs policy decisions aimed at improving healthcare delivery.

Subsequently, an analysis was conducted by consulting comprehensive administrative databases that are continuously updated with data from Community Pharmacies. This data is managed through a sophisticated system developed by IQVIA, a leading provider of healthcare data analytics, which compiles information from various sources, including the Federfarma flow, a network representing pharmacies across Italy. To ensure patient privacy, all data analyzed were presented in aggregate form, which means that individual prescriptions could not be identified, thus maintaining confidentiality while still allowing for the examination of overall trends and patterns in pharmaceutical utilization. This approach enables researchers to derive meaningful insights into medication use without compromising patient anonymity.

The analysis focused on the top 30 active ingredients impacting pharmaceutical expenditure, as indicated in the Osmed Report 2022, the aim was to assess the approach to spending on pharmacological therapies in different macro-areas and any heterogeneity in the use of AIFA Notes, by examining the different use of private purchasing for drugs classified as reimbursement class A. The percentage of use of private purchases in the various macro-areas was analyzed, using the national data as a benchmark.

To assess pharmaceutical expenditure and potential misuse of public resources, we utilized data from the OsMed (Osservatorio Nazionale sull’Impiego dei Medicinali) Report 2022, focusing on regional variations in expenditure for Class A (fully reimbursed) and Class C (non-reimbursed) drugs. Subsequently, an analysis was conducted by consulting comprehensive administrative databases that are continuously updated with data from contracted community pharmacies. This data is managed through a sophisticated system developed by IQVIA, a leading provider of healthcare data analytics, which compiles information from various sources, including the Federfarma flow, a network representing pharmacies across Italy. We analyzed total regional pharmaceutical expenditure, with a particular focus on out-of-pocket spending. While a direct, quantitative analysis of AIFA Note application was not performed, we analyzed the regional differences in the expenditure for drugs that are typically subject to AIFA Notes restrictions, as reported in the OsMed Report. This provided an indirect measure of potential regional variations in AIFA Note utilization. Data on regional income levels were also collected from ISTAT (Istituto Nazionale di Statistica) to examine the relationship between economic status and out-of-pocket spending,

## Results

### Pharmaceutical expenditure in Italy and the role of AIFA in ensuring sustainability and appropriateness

In 2021, the total pharmaceutical expenditure, including both public and private components, amounted to €32.2 billion, representing a 3.5% increase compared to 2020 and accounting for 1.9% of the current GDP. Gross public pharmaceutical expenditure, with a value of €22.3 billion, accounted for 69% of the total pharmaceutical expenditure and increased by 2.6% compared to 2020. Private expenditure, including citizen cost-sharing, amounted to €9.2 billion [15].

One of the variables influencing territorial pharmaceutical expenditure is citizen cost-sharing. Citizen cost-sharing serves as a control tool for drug consumption and expenditure in Italy and consists of two elements: the prescription fee (per prescription or per package), the amount of which varies in different regions, and the amount exceeding the reference price or reimbursement price, which is determined by AIFA (Italian Medicines Agency) based on a transparency list, usually corresponding to the price of the cheapest equivalent drug in a given therapeutic category. The price difference between the cheapest generic (reimbursed by the National Health Service) and the specialty drug is borne by the citizen [16].

The co-payment, introduced by law 405/2001 [17] in the National Health Service, initially served as a tool to promote citizen responsibility, mainly aimed at discouraging excessive consumption of drugs and medical services. However, at the regional level, it has increasingly become a significant source of healthcare funding. Traditional economic theory assigns a dual role to co-payments: first, to control demand, and second, to finance healthcare expenditure. The introduction of patient cost-sharing in pharmaceutical spending aims to finance the National/Regional Health Service while simultaneously reducing overconsumption of healthcare services.

In the realm of consumer governance, the Italian State has granted the Medicines Agency, established by Article 48, Legislative Decree No. 269 of 2003 [18], the discretionary power to compile the list of drugs eligible for reimbursement by the National Health Service based on cost and effectiveness criteria. Moreover, mechanisms for price discounts on reimbursable drugs have been established to contain pharmaceutical expenditure, ensuring the widest possible access to essential drugs listed for reimbursement by the National Health Service. The cost of using drugs for off-label indications, often limited by AIFA’s restrictive notes, is borne by the patient and falls under out-of-pocket expenses.

AIFA’s notes on prescription appropriateness represent a crucial step towards rational and conscious use of drugs reimbursed by the National Health Service. Therefore, their misuse outside of the approved indications represents instances of financial damage to the healthcare system.

The Osmed Report highlights interregional differences in out-of-pocket expenditure. This data can likely be attributed to four factors:

- Difference in per capita GDP;
- Different cultural approaches to the use of generic drugs and biosimilars;
- Inappropriate reliance on AIFA notes for prescribing appropriateness;
- Different regional pharmaceutical and healthcare governance policies.

### The incidence of private purchasing in Italy and the different use of AIFA notes

As highlighted in the Osmed Report 2022 [19], “In 2022 the cost of sharing for the portion exceeding the reference price of expired patent medicines (hereinafter co-participation) was equal to 18.4 euros per capita (around 1.1 billion euros). This value represents 73.1% of the total participation of the citizen (also including the ticket for prescription and/or packaging) and records an increase of 0.1% compared to the previous year and a CAGR (Compound Annual Growth Rate) of + 1.2% starting from 2017. The highest per capita expenditure by co-participation is recorded in the South and in the Islands (23.9 euro), while the smallest one in the North with 13.7 euros, deviating from the national average value of + 30.1% and − 25.4% respectively. Calabria, Lazio and Campania is the Region with the h1ighest expenditure values (respectively 25.6, 25.3 and 25.2 euros), while the PAs of Bolzano and Trento and the Aosta Valley record the highest values low, respectively equal to 12, 13.1 and 12.9 euros.”

A correlation analysis between cost-sharing expenditure and regional per capita income reveals that regions with lower incomes tend to have higher cost-sharing. Notably, Calabria, Campania, Sicily, and Puglia, with per capita incomes slightly below or slightly above €10,000, demonstrate elevated cost-sharing levels compared to the national average (>€20).

**Fig. 1:**
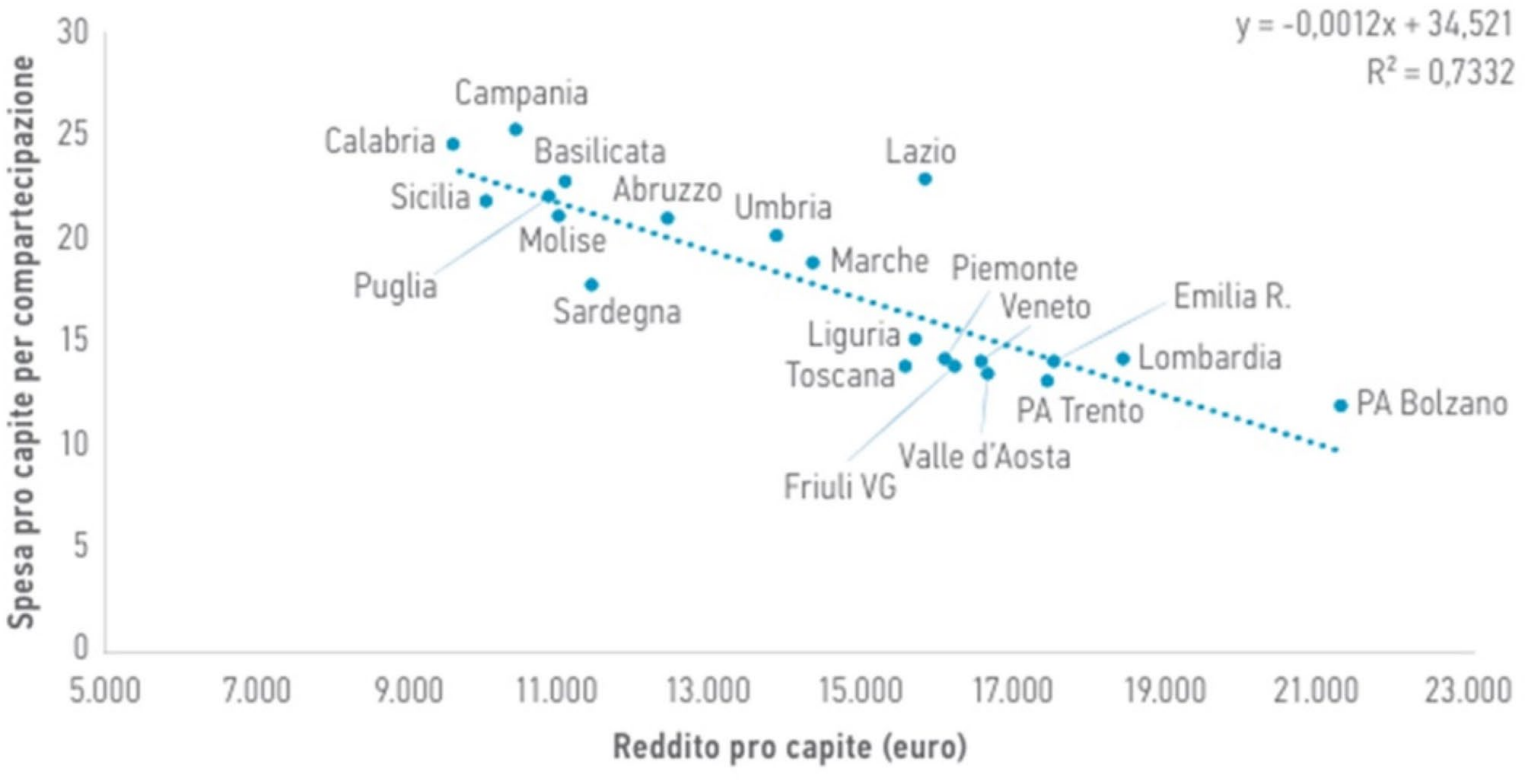
Correlation between per capita income and cost-sharing in pharmaceutical expenditure - Osmed Report “The Use of Drugs in Italy. National Report 2020”. AIFA, re-elaborated by G.L. Colombo et al. in “La relazione tra compartecipazione del paziente alla spesa farmaceutica, aderenza terapeutica e reddito: una revisione di letteratura”

The discussed phenomenon, which involves higher per capita cost-sharing in the South (the price difference between the cheapest generic drug reimbursed by the National Health Service and the specialty drug is borne by the citizen), becomes even more significant when linked to per capita income in different regions. In fact, this correlation reveals that regions with lower per capita income have higher per capita cost-sharing. Specifically, Campania, Calabria, and Sicily, where the average per capita declared income is around €10,000, are noteworthy examples. On the other hand, regions with higher per capita income (Provinces of Trento, Bolzano, Lombardy) have lower cost-sharing. Based on these observations, it becomes evident that cost-sharing is linked to income, but with an inverse proportional relationship (Fig. 1). The lower the declared per capita income, the higher the cost-sharing for the amount exceeding the reference price. There can be multiple causes for this:

- Culture and Preferences: There are cultural factors that influence patients’ choices. In many of these regions, patients may be more inclined to purchase branded drugs rather than equivalents, even if the latter are more economical. This preference leads to an increase in out-of-pocket spending.
- Limitations of the National Health Service (SSN): Low-income regions often face challenges related to the quality and access to healthcare services. Long waiting lists and limited availability of drugs can push citizens to resort to private drugs, thus increasing the cost-sharing expenditure.
- Regional Policies: Cost-sharing policies vary from region to region. Some regions have reintroduced the ticket for pharmaceutical expenses to keep spending under control, which can further influence the amount that citizens have to pay out-of-pocket.

**Table 1:** Composition of total pharmaceutical expenditure by region in 2022. Data source: Osmed report “the use of drugs in Italy. National report 2022”. AIFA. https://www.aifa.gov.it/documents/20142/1967301/Rapporto-OsMed-2022.pdf

Region	Gross agreed expenditure§ 1		Private class A		Class C with prescription		Self-medication		Commercial establishments		Public facilities		Total
	€*	%*	€*	%*	€*	%*	€*	%*	€*	%*	€*	%*	€*
Piedmont	624	25.7	186	7.6	281	11.6	209	8.6	25	1.0	1.107	45.5	2.432
Aosta Valley	17	25.5	10	15.0	7	10.5	7	10.5	1	1.5	25	36.9	67
Lombardy	1.854	33.8	270	4.9	633	11.5	480	8.7	59	1.1	2.196	40.0	5.492
Autonomous Province of Bolzano	57	23.5	12	5.0	23	9.5	28	11.6	0	-	122	50.5	242
Autonomous Province of Trento	76	28.6	17	6.4	26	9.8	29	10.9	2	0.8	116	43.6	266
Veneto	651	25.5	131	5.1	275	10.8	239	9.4	22	0.9	1.237	48.2	2.555
Friuli VG	184	27.7	28	4.2	65	9.8	57	8.6	5	0.8	324	48.9	663
Liguria	241	24.8	61	6.3	123	12.6	92	9.5	10	1.0	446	45.8	973
Emilia R.	588	23.8	95	3.8	263	10.7	215	8.7	35	1.4	1.273	51.6	2.469
Tuscany	530	26.0	90	4.4	235	11.5	187	9.2	28	1.4	970	47.5	2.040
Umbria	141	27.1	27	5.2	57	11.0	36	6.9	5	1.0	253	48.8	519
Marche	246	27.0	47	5.2	95	10.4	67	7.4	7	0.8	448	49.2	910
Lazio	1.034	30.8	261	7.8	358	10.7	273	8.1	20	0.6	1.410	42.0	3.356
Abruzzo	241	28.8	91	10.9	71	8.5	53	6.3	7	0.8	374	44.7	837
Molise	51	32.3	4	2.5	14	8.9	10	6.3	1	0.6	78	49.4	158
Campania	1.044	30.7	171	5.0	358	10.5	256	7.5	40	1.2	1.530	45.0	3.399
Apulia	726	32.1	80	3.5	207	9.1	147	6.5	19	0.8	1.084	47.9	2.263
Basilicata	105	34.5	5	1.6	23	7.5	18	5.9	3	1.0	151	49.5	305
Calabria	352	32.5	50	4.6	98	9.0	62	5.7	10	0.9	511	47.2	1.083
Sicily	838	33.9	156	6.3	220	8.9	137	5.5	13	0.5	1.105	44.7	2.469
Sardinia	282	27.9	116	11.5	90	8.9	60	5.9	14	1.4	447	44.3	1.009
Italy	**9.880**	**29.5**	**1.908**	**5.7**	**3.523**	**10.5**	**2.661**	**7.9**	**326**	**1.0**	**15.208**	**45.4**	**33.506**
North	4.291	28.3	810	5.3	1.696	11.2	1.355	8.9	159	1.0	6.846	45.2	15.157
Central	1.951	28.6	425	6.2	745	10.9	564	8.3	60	0.9	3.081	45.1	6.826
South	3.639	31.6	673	5.8	1.082	9.4	742	6.4	108	0.9	5.281	45.8	11.525

§ The expense refers to class A-SSN drugs and class C drugs (19 million euros) reimbursed by the NHS

* million euros

* calculated on the total regional expenditure

In Table 1, the composition of pharmaceutical expenditure by region is reported, including the breakdown of drugs that, despite being classified in reimbursement class A, are purchased privately. Table 1 presents a regional breakdown of total pharmaceutical expenditure in Italy for 2022, drawing data from the OsMed Report 2022. This overview is vital for understanding our analysis of out-of-pocket (OOP) spending and AIFA Note utilization. By examining the composition of pharmaceutical expenditure—specifically the balance between public and private spending across regions—we can assess the impact of regional disparities and inappropriate AIFA note usage on patients’ financial burdens. This foundational data allows us to investigate how spending variations contribute to inequities in access to essential medications and highlight areas for better allocation of public resources. Importantly, this data contradicts the Osmed report on cost-sharing and cannot simply be correlated with different per capita GDP figures. The southern regions, on average, spend less than the national average on class A drugs without reimbursement and on class C drugs, fully at the citizens’ expense. Thus, it is likely that the increased expenditure in these regions is due to the purchase of branded drugs rather than generics.

Based on this information, an analysis was conducted by consulting the administrative databases fed by the flow of contracted pharmaceuticals and the Federfarma flow to assess the approach to spending on pharmacological therapies in different macro-areas and the possible heterogeneity in the use of AIFA notes.

Table 2 shows the different use of private purchasing for drugs classified as reimbursement class A, specifically analyzing the top 30 active ingredients impacting pharmaceutical expenditure as reported in the Osmed report 2022. It can be observed that the regions in southern Italy have lower private purchasing of cholecalciferol, amoxicillin and clavulanic acid, and certain proton pump inhibitors, while for other active ingredients, private purchasing is in line, if not higher, compared to other areas (e.g., the purchase of heparins). Since these active ingredients are subject to AIFA notes (cholecalciferol and proton pump inhibitors) and strict prescribing appropriateness policies (antibiotics), it is conceivable that the different use of cost-sharing is influenced by cultural factors that favor the purchase of branded products over generics. However, for specific conditions and widely used molecules, this can lead to improper use of resources. It is also important to consider that private purchasing of class A drugs includes any prescriptions for non-reimbursed therapeutic indications or indications not listed in the summary of product characteristics (SmPC). These prescriptions can sometimes contribute to inappropriate usage, which, depending on local and/or regional governance, can impact the usage of drugs subject to AIFA notes in different ways.

**Table 2:** Incidence of private purchasing in 2022 for the top 30 active ingredients impacting contracted pharmaceutical expenditure, as per Table 3 of the latest Osmed report 2022. Data source: IQVIA

		ITALY		MACRO CENTRAL AREA		MACRO NORD AREA		MACRO SUD AREA	
Atc	Active Principle	Ddd X 1000 Inhabitants Private Purchases	% Incidence of Private Purchases	Ddd X 1000 Inhabitants Private Purchases	% Incidence of Private Purchases	Ddd X 1000 Inhabitants Private Purchases	% Incidence of Private Purchases	Ddd X 1000 Inhabitants Private Purchases	% Incidence of Private Purchases
A02BC01	OMEPRAZOLE	43,08	17,2	210,81	18,02	275,11	19,94	356,11	14,88
A02BC02	PANTOPRAZOLO	68,45	16,65	432,93	21,17	528,81	16,14	447,12	15,04
A02BC03	LANSOPRAZOLO	27,99	15,45	152,94	16,93	210,38	11,97	195,12	14,01
A02BC05	ESOMEPRAZOLO	42,6	18,58	212,5	22,82	332,12	16,21	319,5	17,95
A07AA11	RIFAXIMINA	2,91	11,94	17,1	12,83	20,68	12,16	19,03	10,12
A07EC02	MESALAZINA	4,99	7,33	28,36	7,99	31,4	6,08	39,95	7,88
A10AB05	INSULINA ASPART	0,64	1,92	3,51	1,63	1,27	0,55	7,59	2,82
A10BA02	METFORMINA	37,08	11,93	149,63	9,9	279,59	11,83	250,02	10,18
A11CC05	COLECALCIFEROLO	497,18	26,12	2.693,28	31,32	4.905,11	28,06	2.775,67	20,12
B01AB05	ENOXAPARINA	3,07	9,24	10,31	11,65	17,02	10,51	28,16	12,56
C07AB07	BISOPROLOLO	13,4	8,14	53,39	6,85	117,25	7,88	92,79	7,92
C08CA01	AMLODIPINA	24,09	6,79	121,01	5,98	199,51	6,44	149,2	7,18
C09AA05	RAMIPRIL	78,56	9,76	379,39	8,38	597,6	8,42	494,45	10,6
C09CA08	OLMESARTAN MEDOXOMIL	11,99	5,74	58,27	5,65	88,36	5,54	90,43	4,97
C09DX03	OLMESARTAN MEDOXOMIL- AMLODI	0,07	43,75	0,45	47,37	0,63	38,41	0,38	46,91
C10AA01	SIMVASTATINA	13,31	8,98	63,24	8,12	107,89	9,05	73,43	7,11
C10AA05	ATORVASTATINA	23,14	3,71	111,52	3,47	169,86	3,23	170,4	3,88
C10AA07	ROSUVASTATINA	21,79	10,48	126,92	11,29	140,83	8,1	153,79	11,35
C10AX06	OMEGA-3-TRIGLICERIDI INCLUSI	4,33	12,63	23,17	12,67	38,57	16,38	26,51	9,39
J01CR02	AMOXICILLINA ED INIBITORE DELLE LATTAMASI	25,14	29	118,54	26,59	214,78	31,57	152,7	23
L02BG04	LETROZOLO	0,92	4,07	4,82	4,5	3,45	1,7	9,43	5,16
N02AB03	FENTANIL	0,55	7,02	2,82	7,16	4,43	5,29	3,98	7,39
N02BF02	PREGABALIN	3,49	10,82	18,55	10,79	31	10,16	19,9	9,57
N03AX14	LEVETIRACETAM	2,07	7,18	11,67	8,1	12,93	5,25	14,35	6
R03AK07	FORMOTEROLO E BUDESONIDE	1,38	5,58	6,41	5,71	9,95	4,44	12,5	7,73
R03AK08	FORMOTEROLO E BECLOMETASONE	2,39	4,76	12,35	4,51	14,04	3	25,85	8,49
R03AK10	VILANTEROLO E FLUTICASONE FU	0,71	1,64	4,28	1,76	4,62	1,41	6,99	2,17

DDD = Defined Daily Dose (source. https://www.aifa.gov.it/documents/20142/1967301/Rapporto-OsMed-2022.pdf)

Indeed, considering the detailed out-of-pocket data for AIFA notes (Supplementary Table 1), a substantial difference can be observed in the use of AIFA notes across different macro-areas compared to the Italian average. This difference cannot be explained solely by variations in disease incidence, considering that this increase involves different categories of drugs. There is a higher utilization in the southern regions of AIFA Note 8 (levocarnitine), 15 (human albumin), 66 (anti-inflammatory drugs), 87 (oxybutynin), 88 (corticosteroids in dermatological preparations), and 96 (cholecalciferol and vitamin D analogs). In several notes like Note 31 (Levodropropizine, Dihydrocodeine Benzoic Acid, Dihydrocodeine), Note 55 (penicillins, cephalosporins and aminoglycosides), Note 56 (Teicoplanin, Rifabutin, Imipenem + Cilastatin, Aztreonam), Note 66 (NSAID drugs), Note 83 (medications for the topical treatment of xerophthalmia), Note 84 (Valacyclovir, Famciclovir, Brivudine, Acyclovir), Note 89 (antihistamines), Note 95 (drugs for actinic keratosis) and Note 97 (vitamin K antagonist and NAO drugs), the consumption value of the southern regions is slightly higher than the Italian areas.

In particular, Note 1 and Note 48, although including the same drugs, are adopted for different settings. Therefore, in this study, they were also analyzed cumulatively.

On the other hand, there is significantly lower utilization of Note 39 (growth hormone and analogs like somatropin and somatropin) compared to other areas and the Italian average. The data compares the value of drugs purchased by contracted pharmacies (sell-in) with the amount reimbursed (sell-out). This ratio is of great importance as inappropriate reliance on restrictive notes at the time of prescription constitutes a case of financial damage. Furthermore, comparing it with the national benchmark allows for the verification of any deviations from the average prescribing behavior and the use of AIFA notes by General Practitioners in different geographical areas.

**Table 3:** Regional disparities in pharmaceutical access and expenditure: key drivers

Macro Area	Average Income (€)	OOP Pharmaceutical procapita Spending (€)	AIFA Notes Exceeding National Average (Coverage Data)	Key Observations
Northern Regions	30,000	13,7	1, 1/48, 28, 36, 48, 51, 74, 75, 82, 85, 90, 92, 93, 97,99, 100	Higher income, lower OOP spending. Presumed greater general AIFA Note utilization contributes to lower OOP costs, but cultural differences play a role in brand-name preferences.
Central Regions	25,000	20,3	39, 42, 65	Moderate income, moderate OOP spending. Mixed adherence to AIFA Notes contributes to intermediate OOP costs. Reimbursement criteria have an impact, cultural differencs may play a small role
Southern Regions	18,000	23,9	2, 8, 13, 15, 31, 55, 56, 66, 79, 83, 84, 87, 88, 89, 91, 95, 96	Lower income, higher OOP spending. Inappropriate or less restrictive use of certain AIFA Notes, contributes to high OOP costs, coupled with cultural preferences for brand-name drugs and potential information gaps.

The use of AIFA notes varies across different contexts, highlighting a limitation of this governance tool. While it has helped align regulatory decisions with Evidence-Based Medicine over the years, it cannot fully replace or integrate Clinical Practice Guidelines (CPGs). This is because it does not capture the clinical complexity and social aspects of care. For instance, the higher use of AIFA Note 66 in the southern macro-area may be linked to the non-reimbursability of the first-choice active ingredient, as indicated by major CPGs, in the treatment of osteoarthritic pain. However, this relationship must be understood in the context of regional governance policies, which may influence the delivery of certain active ingredients. Therefore, while AIFA notes have their role, they are not a comprehensive solution and must be considered within the broader healthcare governance framework (e.g., AIFA Notes 36, 74, 75, 85, 97).

## Discussion

Out-of-pocket expenditure in healthcare is a complex issue with significant financial and health implications. High OOP costs can create barriers to accessing essential medical services, exacerbate health disparities, and lead to financial distress for individuals and families. Policymakers should consider a combination of approaches, including universal healthcare, insurance reforms, income-based subsidies, and improved price transparency, to address this pressing problem and ensure equitable access to healthcare for all. Access to sustainable healthcare and universal care are common values on which the National Health Service is based, despite the organizational and financial heterogeneity of individual regions. The chronicity of diseases and population aging inevitably lead to increased costs of technologies, the need for innovative therapies, difficulties in recruiting personnel to ensure a homogeneous distribution throughout the healthcare sector, and the need to maintain healthcare expenditure sustainability. These factors result in inequalities in the population in terms of health outcomes, starting from access to services [20].

A prior Japanese study found that there is no evidence that reduced cost-sharing improved health outcomes among middle- and higher-income individuals, but found that it significantly improved self-reported health among lower-income individuals [21].

Moreover, initiatives to improve price transparency empower patients to make informed decisions about their healthcare, potentially leading to cost savings and a reduction in OOP expenses. When individuals are equipped with knowledge about the costs of various treatment options, they can actively engage in managing their healthcare costs, further enhancing their financial well-being [22]. The data presented in the chapter dedicated to private healthcare consumption in the OASI 2022 [12] report by CERGAS (Centre for Research on Health and Social Care Management) highlights that the percentage of fully paid services (out-of-pocket or with total or partial reimbursement) exceeds 40% for specialist visits and over 25% for instrumental diagnostic tests. The phenomenon of out-of-pocket payments, which constitutes approximately 23% of total per capita healthcare expenditure, highlights a significant concern regarding the misuse of pharmaceutical expenditure. When individuals are required to cover a substantial portion of their healthcare costs directly, it can lead to financial strain and potentially result in the inappropriate use of medications. This situation is exacerbated by the limited role of private intermediary spending, which accounts for only around 3% of healthcare financing, indicating that the burden of healthcare costs largely falls on individuals.

As a result, patients may resort to suboptimal purchasing decisions, such as opting for cheaper, potentially less effective medications or foregoing necessary treatments altogether due to cost concerns. This misuse can undermine the overall effectiveness of the healthcare system and compromise patient outcomes. Therefore, it is essential to address the implications of out-of-pocket payments on pharmaceutical expenditure to ensure that healthcare remains equitable and accessible for all individuals.

In this research, we found that for many drugs subjected to AIFA notes, their use is heterogeneous across Italy. The inappropriate reliance on restrictive notes at the time of prescription constitutes a case of financial damage, as it indicates a high level of out-of-pocket spending by patients for these drugs. This ratio is of great importance in assessing the extent of this issue.

Furthermore, comparing this ratio with the national benchmark allows for the verification of any deviations from the average prescribing behavior and the use of AIFA notes by General Practitioners in different geographical areas. This analysis can help identify regions or areas where there is a higher-than-average use of restrictive notes, which may lead to an increased financial burden on patients and potentially inappropriate prescribing practices.

Interestingly, the regions where there is a more inappropriate use of AIFA notes are the same ones with the highest out-of-pocket spending and the lowest income. This observation leads us to hypothesize that there is a different perception regarding the out-of-pocket purchase of medications. In Southern regions when is possible to purchase drugs under full reimbursement, public funds are used, even improperly, while there is a choice to use branded drugs instead of generics, which seems to be fundamentally based on cultural aspects rather than economic considerations.

If patients are aware of and accountable for the costs associated with medications and healthcare services, they may become more selective in their choices, opting out of treatments with little evidence of effectiveness and choosing less expensive prescription drugs when given the option [23].

Furthermore, in recent years, characterized by the coexistence of crisis factors such as the COVID-19 pandemic, geopolitical changes (such as the Russo-Ukrainian conflict), inflation and energy cost escalation, and disruption of supply chains, the analysis of the relationship between population health and the country’s economic growth has become increasingly important [24]. While in the past it was widely recognized that populations in high-income countries enjoyed higher levels of health and longer life expectancies, in recent years, researchers have begun to study the inverse relationship: a higher level of health positively impacts a country’s ability to generate income and economic growth. In this context, it is evident that the use of healthcare cost-sharing depends on multiple factors, and cultural and economic factors influence the approach to the proper use of public resources [25]. Policymakers should consider this increase as an indicator of poor quality and difficulty in accessing care. So, there is an urgent need for greater policy and research efforts to improve the financial security of people living with chronic health conditions and disabilities. This can be achieved, in part, by ensuring they have access to comprehensive health insurance coverage by reallocating resources currently in use [26]. An important limitation of this study is the provenance of the data because Federfarma provides private flows and public ones are necessary for a complete analysis. In addition, the data are aggregated and therefore cannot be traced back to the individual user and his clinical situation. This study proposes, therefore, a simple analysis of the behaviors related to the use of drugs in the population.

While this study reveals regional variations in AIFA Note application and their impact on out-of-pocket spending, it did not explicitly model the influence of clinical and epidemiological factors. Future research should explore how disease prevalence, patient characteristics, and regional policies interact with AIFA Note implementation to affect patient costs and access to medications. Studies incorporating patient-level clinical data and qualitative research on provider and patient perspectives are needed to provide a more nuanced understanding of these complex relationships and inform more equitable healthcare policies.

## Conclusions

The trend toward reliance on private funding or, worse, the improper use of healthcare resources could undermine equity and threaten the legitimacy of the public healthcare system. It is essential to recognize that private consumption is not a homogeneous entity. While it accounts for approximately a quarter of total healthcare expenditure, this observation alone is insufficient. It is crucial to analyze its individual components and the complex relationships linking them to public consumption, employing new conceptual frameworks that differ from those traditionally used. In this context, the present study provides data and evidence on a phenomenon that is increasingly the subject of debate, although often lacking adequate supporting information.

Therefore, policies aimed at facilitating access and improving public healthcare, along with proper allocation of public resources, should be considered as tools to reduce inequalities. National health objectives can only be achieved by ensuring accessibility of healthcare services, increasing public healthcare spending correctly, and monitoring national spending programs. It is also important to raise awareness among the population about this issue, with a clear focus on individual patients, emphasizing patient involvement in the treatment process, with the goal of enabling patients to better manage their conditions. Furthermore, strong moral suasion regarding the proper use of AIFA notes would be desirable since they represent an important step towards rational and conscious use of drugs reimbursed by the National Health Service. Other forms of healthcare autonomy risk amplifying regional inequalities and undermining national governance tools at a time when the reorganization of healthcare services is linked to the use of resources from the National Recovery and Resilience Plan (PNRR) and requires reducing regional disparities.

## Electronic supplementary material

Below is the link to the electronic supplementary material.

Supplementary Material 1

## Data Availability

Data is provided within the manuscript or supplementary information files.
