# Supplementary material for: Out-of-pocket pharmaceutical expenditure and potential misuse of public resources - analysis in the Italian context
Source: Cost Eff Resour Alloc. 2025 Apr 17;23:17. doi: 10.1186/s12962-025-00619-7 (PMC12007333; doi:10.1186/s12962-025-00619-7)
Supplement: Supplementary file 1 — Supplementary Material 1 [file 12962_2025_619_MOESM1_ESM.docx]

Supplementary Table 1 - Utilization of AIFA notes in 2022. The data is compared with other geographies. Data source: IQVIA.

| **AIFA NOTES** | **GEOGRAPHY** | **SELLIN VALUE YEAR 2022 (€)** | **SELLOUT VALUE REFUNDED YEAR 2022 (€)** | **Use % NOTE AIFA** |
| --- | --- | --- | --- | --- |
|  |  |  |  |  |
| **NOTE 1** | **ITALY** | **652.217,390** | **285.421,070** | **43,76** |
|  | MACRO CENTRAL AREA | 150.261,320 | 70.157,340 | 46,69 |
|  | MACRO NORTH AREA | 230.337,360 | 123.774,200 | 53,74 |
|  | MACRO SOUTH AREA | 271.618,710 | 91.489,530 | 33,68 |
| **NOTE 2** | **ITALY** | **67.171.932,780** | **54.330.843,530** | **80,88** |
|  | MACRO CENTRAL AREA | 15.989.578,360 | 12.910.906,440 | 80,75 |
|  | MACRO NORTH AREA | 24.886.280,020 | 20.093.745,650 | 80,74 |
|  | MACRO SOUTH AREA | 26.296.074,650 | 21.326.191,300 | 81,10 |
| **NOTE 4** | **ITALY** | **165.358.102,980** | **147.312.066,270** | **89,09** |
|  | MACRO CENTRAL AREA | 41.365.782,660 | 36.867.579,840 | 89,13 |
|  | MACRO NORTH AREA | 79.535.737,850 | 70.863.949,450 | 89,10 |
|  | MACRO SOUTH AREA | 44.456.582,610 | 39.580.536,630 | 89,03 |
| **NOTE 8** | **ITALY** | **2.478.919,410** | **345.699,090** | **13,95** |
|  | MACRO CENTRAL AREA | 567.584,820 | 59.168,340 | 10,42 |
|  | MACRO NORTH AREA | 1.053.268,920 | 108.737,640 | 10,32 |
|  | MACRO SOUTH AREA | 858.065,670 | 177.793,110 | 20,72 |
| **NOTE 1 / NOTE 48** | **ITALY** | **779.183.383,260** | **645.297.764,590** | **82,82** |
|  | MACRO CENTRAL AREA | 168.512.870,780 | 136.607.440,900 | 81,07 |
|  | MACRO NORTH AREA | 325.590.280,580 | 270.075.145,340 | 82,95 |
|  | MACRO SOUTH AREA | 285.080.231,480 | 238.615.178,110 | 83,70 |
| **NOTE 13** | **ITALY** | **973.351.579,380** | **896.099.503,110** | **92,06** |
|  | MACRO CENTRAL AREA | 230.230.696,850 | 211.748.969,700 | 91,97 |
|  | MACRO NORTH AREA | 412.694.160,770 | 379.805.414,290 | 92,03 |
|  | MACRO SOUTH AREA | 330.426.720,550 | 304.545.116,910 | 92,17 |
| **NOTE 15** | **ITALY** | **14.212.574,390** | **13.734.534,360** | **96,64** |
|  | MACRO CENTRAL AREA | 2.175.785,190 | 2.028.147,980 | 93,21 |
|  | MACRO NORTH AREA | 2.212.482,370 | 2.082.889,550 | 94,14 |
|  | MACRO SOUTH AREA | 9.824.306,840 | 9.623.496,980 | 97,96 |
| **NOTE 28** | **ITALY** | **3.013.889,810** | **2.507.118,150** | **83,19** |
|  | MACRO CENTRAL AREA | 694.007,970 | 573.365,400 | 82,62 |
|  | MACRO NORTH AREA | 1.085.820,040 | 916.966,020 | 84,45 |
|  | MACRO SOUTH AREA | 1.234.061,800 | 1.016.786,720 | 82,39 |
| **NOTE 31** | **ITALY** | **14.031.763,120** | **1.038.705,000** | **7,40** |
|  | MACRO CENTRAL AREA | 2.991.672,400 | 246.463,360 | 8,24 |
|  | MACRO NORTH AREA | 7.855.182,760 | 498.350,040 | 6,34 |
|  | MACRO SOUTH AREA | 3.184.908,090 | 293.891,600 | 9,23 |
| **NOTE 36** | **ITALY** | **1.008.688,430** | **248.648,520** | **24,65** |
|  | MACRO CENTRAL AREA | 223.550,870 | 39.514,760 | 17,68 |
|  | MACRO NORTH AREA | 368.624,760 | 135.403,800 | 36,73 |
|  | MACRO SOUTH AREA | 416.512,800 | 73.729,960 | 17,70 |
| **NOTE 39** | **ITALY** | **3.915.793,560** | **2.206.053,650** | **56,34** |
|  | MACRO CENTRAL AREA | 1.179.697,600 | 924.039,730 | 78,33 |
|  | MACRO NORTH AREA | 1.785.600,430 | 1.049.155,560 | 58,76 |
|  | MACRO SOUTH AREA | 950.495,560 | 232.858,380 | 24,50 |
| **NOTE 42** | **ITALY** | **751.885,220** | **334.536,520** | **44,49** |
|  | MACRO CENTRAL AREA | 161.681,510 | 74.589,110 | 46,13 |
|  | MACRO NORTH AREA | 225.341,950 | 99.919,360 | 44,34 |
|  | MACRO SOUTH AREA | 364.861,760 | 160.028,050 | 43,86 |
| **NOTE 48** | **ITALY** | **28.762.945,310** | **21.397.854,420** | **74,39** |
|  | MACRO CENTRAL AREA | 5.313.435,090 | 3.844.336,590 | 72,35 |
|  | MACRO NORTH AREA | 13.573.919,350 | 10.394.595,700 | 76,58 |
|  | MACRO SOUTH AREA | 9.875.590,990 | 7.158.922,070 | 72,49 |
| **NOTE 51** | **ITALY** | **4.872.430,340** | **1.825.477,700** | **37,47** |
|  | MACRO CENTRAL AREA | 943.650,900 | 286.137,680 | 30,32 |
|  | MACRO NORTH AREA | 2.551.826,690 | 1.086.666,890 | 42,58 |
|  | MACRO SOUTH AREA | 1.376.952,770 | 452.673,130 | 32,87 |
| **NOTE 55** | **ITALY** | **17.297.983,760** | **15.404.266,420** | **89,05** |
|  | MACRO CENTRAL AREA | 4.008.457,120 | 3.522.493,710 | 87,88 |
|  | MACRO NORTH AREA | 1.950.181,420 | 1.556.635,190 | 79,82 |
|  | MACRO SOUTH AREA | 11.339.345,130 | 10.325.137,510 | 91,06 |
| **NOTE 56** | **ITALY** | **3.154.258,610** | **3.703.797,690** | **117,42** |
|  | MACRO CENTRAL AREA | 956.392,070 | 1.096.886,250 | 114,69 |
|  | MACRO NORTH AREA | 343.015,060 | 398.792,870 | 116,26 |
|  | MACRO SOUTH AREA | 1.854.851,480 | 2.208.118,590 | 119,05 |
| **NOTE 65** | **ITALY** | **131.655,630** | **139.852,590** | **106,23** |
|  | MACRO CENTRAL AREA | 13.750,320 | 16.887,110 | 122,81 |
|  | MACRO NORTH AREA | 9.184,980 | 6.861,030 | 74,70 |
|  | MACRO SOUTH AREA | 108.720,330 | 116.104,450 | 106,79 |
| **NOTE 66** | **ITALY** | **319.526.890,580** | **137.694.662,380** | **43,09** |
|  | MACRO CENTRAL AREA | 72.807.770,780 | 32.464.954,490 | 44,59 |
|  | MACRO NORTH AREA | 130.043.671,790 | 45.179.734,840 | 34,74 |
|  | MACRO SOUTH AREA | 116.675.448,750 | 60.049.973,210 | 51,47 |
| **NOTE 74** | **ITALY** | **10.277.606,620** | **7.616.171,540** | **74,10** |
|  | MACRO CENTRAL AREA | 1.184.143,320 | 399.576,780 | 33,74 |
|  | MACRO NORTH AREA | 7.800.853,680 | 6.460.005,630 | 82,81 |
|  | MACRO SOUTH AREA | 1.292.609,630 | 756.589,100 | 58,53 |
| **NOTE 75** | **ITALY** | **14.830.645,500** | **5.466.508,510** | **36,86** |
|  | MACRO CENTRAL AREA | 2.667.126,840 | 671.662,580 | 25,18 |
|  | MACRO NORTH AREA | 8.927.004,200 | 3.807.502,310 | 42,65 |
|  | MACRO SOUTH AREA | 3.236.514,470 | 987.343,600 | 30,51 |
| **NOTE 79** | **ITALY** | **156.047.085,730** | **139.525.889,170** | **89,41** |
|  | MACRO CENTRAL AREA | 40.621.520,580 | 35.547.086,420 | 87,51 |
|  | MACRO NORTH AREA | 65.814.947,460 | 59.194.400,600 | 89,94 |
|  | MACRO SOUTH AREA | 49.610.617,630 | 44.784.401,840 | 90,27 |
| **NOTE 82** | **ITALY** | **29.681.659,420** | **26.590.239,690** | **89,58** |
|  | MACRO CENTRAL AREA | 7.116.065,760 | 6.373.019,500 | 89,56 |
|  | MACRO NORTH AREA | 11.491.386,160 | 10.507.418,460 | 91,44 |
|  | MACRO SOUTH AREA | 11.074.207,500 | 9.709.801,730 | 87,68 |
| **NOTE 83** | **ITALY** | **1.124.220,520** | **681.997,310** | **60,66** |
|  | MACRO CENTRAL AREA | 267.107,540 | 158.574,900 | 59,37 |
|  | MACRO NORTH AREA | 536.239,860 | 311.353,010 | 58,06 |
|  | MACRO SOUTH AREA | 320.873,110 | 212.069,400 | 66,09 |
| **NOTE 84** | **ITALY** | **36.157.727,950** | **30.180.153,620** | **83,47** |
|  | MACRO CENTRAL AREA | 7.602.403,580 | 6.089.664,130 | 80,10 |
|  | MACRO NORTH AREA | 17.207.982,400 | 14.227.634,240 | 82,68 |
|  | MACRO SOUTH AREA | 11.347.341,840 | 9.862.855,320 | 86,92 |
| **NOTE 85** | **ITALY** | **19.525.027,170** | **12.080.402,200** | **61,87** |
|  | MACRO CENTRAL AREA | 3.065.617,860 | 1.301.640,830 | 42,46 |
|  | MACRO NORTH AREA | 10.381.846,880 | 8.023.250,520 | 77,28 |
|  | MACRO SOUTH AREA | 6.077.562,440 | 2.755.510,830 | 45,34 |
| **NOTE 87** | **ITALY** | **8.533.281,500** | **4.956.962,640** | **58,09** |
|  | MACRO CENTRAL AREA | 2.115.231,840 | 1.270.325,940 | 60,06 |
|  | MACRO NORTH AREA | 4.467.236,180 | 2.446.870,620 | 54,77 |
|  | MACRO SOUTH AREA | 1.950.813,480 | 1.239.766,060 | 63,55 |
| **NOTE 88** | **ITALY** | **25.309.894,490** | **8.357.781,350** | **33,02** |
|  | MACRO CENTRAL AREA | 5.934.991,960 | 1.717.925,450 | 28,95 |
|  | MACRO NORTH AREA | 9.598.205,300 | 2.484.315,440 | 25,88 |
|  | MACRO SOUTH AREA | 9.776.697,180 | 4.155.540,440 | 42,50 |
| **NOTE 89** | **ITALY** | **109.320.973,330** | **63.110.011,560** | **57,73** |
|  | MACRO CENTRAL AREA | 24.872.846,920 | 14.386.948,940 | 57,84 |
|  | MACRO NORTH AREA | 43.479.721,750 | 22.907.125,170 | 52,68 |
|  | MACRO SOUTH AREA | 40.968.404,470 | 25.815.937,350 | 63,01 |
| **NOTE 90** | **ITALY** | **5.785.712,040** | **5.173.202,240** | **89,41** |
|  | MACRO CENTRAL AREA | 1.369.796,200 | 1.202.084,320 | 87,76 |
|  | MACRO NORTH AREA | 2.854.441,060 | 2.605.991,550 | 91,30 |
|  | MACRO SOUTH AREA | 1.561.474,830 | 1.365.126,400 | 87,43 |
| **NOTE 91** | **ITALY** | **33.637.139,890** | **32.020.564,890** | **95,19** |
|  | MACRO CENTRAL AREA | 7.258.098,170 | 6.872.016,020 | 94,68 |
|  | MACRO NORTH AREA | 12.126.495,720 | 11.547.402,690 | 95,22 |
|  | MACRO SOUTH AREA | 14.252.546,110 | 13.601.145,950 | 95,43 |
| **NOTE 92** | **ITALY** | **451.278,750** | **55.932,500** | **12,39** |
|  | MACRO CENTRAL AREA | 97.272,500 | 2.876,250 | 2,96 |
|  | MACRO NORTH AREA | 215.848,750 | 43.290,000 | 20,06 |
|  | MACRO SOUTH AREA | 138.157,500 | 9.766,250 | 7,07 |
| **NOTE 93** | **ITALY** | **2.832.735,460** | **2.396.307,160** | **84,59** |
|  | MACRO CENTRAL AREA | 924.272,540 | 777.042,100 | 84,07 |
|  | MACRO NORTH AREA | 829.807,550 | 737.366,800 | 88,86 |
|  | MACRO SOUTH AREA | 1.078.655,350 | 881.898,250 | 81,76 |
| **NOTE 95** | **ITALY** | **10.245.545,890** | **7.273.355,360** | **70,99** |
|  | MACRO CENTRAL AREA | 2.893.587,690 | 2.017.766,730 | 69,73 |
|  | MACRO NORTH AREA | 4.162.480,480 | 2.787.940,400 | 66,98 |
|  | MACRO SOUTH AREA | 3.189.477,740 | 2.467.648,200 | 77,37 |
| **NOTE 96** | **ITALY** | **333.428.270,630** | **259.265.216,630** | **77,76** |
|  | MACRO CENTRAL AREA | 65.743.109,420 | 47.719.949,640 | 72,59 |
|  | MACRO NORTH AREA | 154.983.154,780 | 117.557.839,540 | 75,85 |
|  | MACRO SOUTH AREA | 112.702.006,800 | 93.987.427,910 | 83,39 |
| **NOTE 97** | **ITALY** | **37.536.483,980** | **29.953.729,460** | **79,80** |
|  | MACRO CENTRAL AREA | 4.414.918,310 | 2.802.836,040 | 63,49 |
|  | MACRO NORTH AREA | 27.975.028,770 | 23.662.035,780 | 84,58 |
|  | MACRO SOUTH AREA | 5.146.537,020 | 3.488.857,670 | 67,79 |
| **NOTE 99** | **ITALY** | **614.015.803,260** | **586.469.575,010** | **95,51** |
|  | MACRO CENTRAL AREA | 149.150.751,390 | 142.257.130,430 | 95,38 |
|  | MACRO NORTH AREA | 264.752.122,530 | 256.619.134,360 | 96,93 |
|  | MACRO SOUTH AREA | 200.112.930,160 | 187.593.311,100 | 93,74 |
| **NOTE 100** | **ITALY** | **215.473.437,530** | **200.295.332,630** | **92,96** |
|  | MACRO CENTRAL AREA | 6.040.866,070 | 2.297.082,770 | 38,03 |
|  | MACRO NORTH AREA | 201.971.170,580 | 193.975.831,580 | 96,04 |
|  | MACRO SOUTH AREA | 7.461.400,310 | 4.022.418,050 | 53,91 |
